# The Effect of Acclydine in Chronic Fatigue Syndrome: A Randomized Controlled Trial

**DOI:** 10.1371/journal.pctr.0020019

**Published:** 2007-05-18

**Authors:** Gerard K. H The, Gijs Bleijenberg, Jos W. M van der Meer

**Affiliations:** Department of General Internal Medicine, Nijmegen Expert Centre Chronic Fatigue, Radboud University Nijmegen Medical Centre, Nijmegen, The Netherlands

## Abstract

**Objectives:**

It is unclear whether insulin-like growth factor (IGF) function is involved in the pathophysiology of chronic fatigue syndrome (CFS). Unpublished data and reports in patient organization newsletters suggest that Acclydine, a food supplement, could be effective in the treatment of CFS by increasing biologically active IGF1 levels. Here we aimed to measure the IGF1 and IGF binding protein (IGFBP) 3 status of CFS patients compared to age- and gender-matched neighborhood controls, and to assess the effect of Acclydine on fatigue severity, functional impairment, and biologically active IGF1 level (IGFBP3/IGF1 ratio).

**Design:**

A randomized, placebo-controlled, double-blind clinical trial.

**Setting:**

Radboud University Nijmegen Medical Centre, The Netherlands.

**Participants:**

Fifty-seven adult patients who fulfilled the US Centers for Disease Control and Prevention criteria for CFS. IGF status of 22 CFS patients was compared to that of 22 healthy age- and gender-matched neighborhood control individuals.

**Intervention:**

Acclydine or placebo for 14 wk.

**Outcome measures:**

Outcomes were fatigue severity (Checklist Individual Strength, subscale fatigue severity [CIS-fatigue]), functional impairment (Sickness Impact Profile-8 [SIP-8]), and biologically active IGF1 serum concentrations. Analyses were on an intention-to-treat basis.

**Results:**

There was no difference in IGF status in 22 CFS patients compared to healthy age- and gender-matched control individuals. Treatment with Acclydine did not result in significant differences compared with the placebo group on any of the outcome measures: CIS-fatigue +1.1 (95% CI −4.4 to +6.5, *p =* 0.70), SIP-8 +59.1 (95% CI −201.7 to +319.8, *p =* 0.65), and IGFBP3/IGF1 ratio −0.5 (95% CI −2.8 to +1.7, *p =* 0.63).

**Conclusion:**

We found no differences in IGF1 status in CFS patients compared to healthy matched neighborhood controls. In addition, the results of this clinical trial do not demonstrate any benefit of Acclydine over placebo in the treatment of CFS.

## Editorial Commentary

**Background:** Chronic fatigue syndrome is a complicated and poorly understood illness. People with the condition experience tiredness that carries on for a long period of time and does not get better with rest. Other symptoms include sleeping problems, muscle and joint pains, concentration difficulties, and headaches. The causes of chronic fatigue syndrome are not known. There is evidence for the effectiveness of certain behavioral interventions, such as exercise therapy and cognitive behavioral therapy, in improving certain symptoms. However, some doctors are concerned that a food supplement called Acclydine, derived from a plant called *Solanum dulcamara,* is being used and promoted as a treatment for chronic fatigue syndrome when there is little evidence about the efficacy or safety of this supplement.

The researchers here carried out a randomized trial in The Netherlands, recruiting adult patients who met the internationally accepted criteria for chronic fatigue syndrome. The participants were allocated by chance to receive either 14 weeks of treatment with Acclydine together with amino acid supplements or, alternatively, placebo versions of the Acclydine and amino acid tablets. The researchers then measured participants' responses with respect to two primary outcome measures. One of these was how tired participants felt, which was measured using a subscale on a questionnaire called the Checklist Individual Strength scale (CIS-fatigue). The other primary outcome measure was “functional impairment,” which examines how well someone carries out their daily life, using the Sickness Impact Profile (SIP-8) scale. The secondary outcome measures included physical activity levels, day-to-day fatigue levels, and the levels of insulin-like growth factor 1 (IGF1) in blood, a hormone that's thought by some researchers to be related to the severity of chronic fatigue.

**What the trial shows:** In the trial, 57 people were randomized to receive either Acclydine plus amino acid supplements, or placebo tablets. When comparing scores on the primary outcome measures at the end of the trial, the researchers did not see significant differences between the treatment and placebo groups. Similarly, the results for secondary outcome measures in this trial did not show any significant differences between the treatment and placebo groups.

**Strengths and limitations:** Strengths of this study include the fact that it is one of the few properly designed studies of a product for which claims have been made of effectiveness in chronic fatigue syndrome. The study was designed as a double-blind trial, in which participants and investigators (those collecting the outcome data) did not know whether an individual received Acclydine or placebo. This procedure should help to minimize bias in assessing outcomes. A key limitation of this study is the relatively small number of participants. However, the investigators considered it unlikely that a larger trial would detect a clinically meaningful effect of Acclydine on these outcome measures for patients with chronic fatigue syndrome.

**Contribution to the evidence:** Systematic reviews of interventions for the treatment of chronic fatigue syndrome have found evidence for the efficacy of exercise therapy and cognitive behavioral therapy. This trial shows no evidence of efficacy of Acclydine plus amino acid supplements for the treatment of chronic fatigue.

## INTRODUCTION

Chronic fatigue syndrome (CFS) is a medically unexplained syndrome, characterized by severe disabling fatigue for a period of at least 6 mo that has led to considerable impairment in daily functioning [1]. Various accompanying symptoms may be present, such as headache, joint and muscle pain, sore throat, and impaired memory and concentration. Of the many therapeutic interventions that have been undertaken, so far only cognitive behavioral therapy and graded exercise therapy have met with success [2].

Neuroendocrinological investigations have tried to elucidate the pathophysiology of CFS [3]. As CFS patients and adult patients with a growth hormone (GH) deficiency report similar symptoms such as fatigue, myalgia, a diminished sense of well-being, and reduced physical capacity [4,5] and treatment of GH-deficient adults with GH has measurable effects on physical function and perception of fatigue [6], GH status is a focus of this research.. Changes in the GH/insulin-like growth factor (IGF) 1 axis have been reported in CFS and fatigue-related disorders such as fibromyalgia [7]. IGF1 studies in CFS patients have yielded conflicting results: low [8,9], normal [10,11], and increased [12] basal IGF1 levels have been found. Sample size, selection of controls, appropriateness of matching, and selection of CFS patients with psychiatric co-morbidity could explain the conflicting results.

Despite the conflicting publications concerning IGF1 status in CFS patients, in recent years there have been reports in patient organization newsletters that a new food supplement called Acclydine could be an effective treatment for CFS. The active ingredient of Acclydine is an alkaloid from *Solanum dulcamara.* It has been claimed that Acclydine is effective because it increases IGF1 concentrations in CFS patients by stimulating GH releasing hormone and, consequently, GH secretion. GH is converted to IGF1 in the liver. IGF1 activates tyrosine kinase and integrin receptors and stimulates intracellular lipid and glycogen synthesis.

Levels of IGF binding proteins (IGFBPs), particularly IGFBP3, modulate the biological activity of IGF1, and the ratio of IGFBP3 to IGF1 reflects IGF1 biological activity [13]. There are unpublished observations suggesting that Acclydine could increase the IGF1 plasma concentrations in healthy humans and in CFS patients. An 8-wk controlled trial with Acclydine in combination with amino acid supplementation in 90 CFS patients has been reported to have a positive effect [14].

However, there are, to our knowledge, no published peer-reviewed studies investigating the effect of Acclydine. Double-blind randomized controlled trials are needed, for several reasons. First, this food supplement is available on the Internet without prescription and is thus easily accessible. Second, although the effects of Acclydine treatment have not been evaluated thoroughly, there are ongoing studies in other vulnerable patient categories, such as in cancer patients with chronic fatigue after treatment, but these studies are not listed in the international controlled trial registry Current Controlled Trials (http://www.controlled-trials.com).

For the reasons given above, we assessed the IGFBP3/IGF1 status in patients fulfilling the US Centers for Disease Control and Prevention (CDC) criteria for CFS [1] and in healthy gender- and age-matched neighborhood controls, to evaluate whether there are intrinsic differences in IGFBP3/IGF1 status between the two groups. Furthermore, we investigated the effect of Acclydine on IGF1 concentration, IGFBP3/IGF1 ratio, and CFS-related symptoms in a well-defined CFS population using validated outcome measures and a randomized double-blind placebo-controlled design.

## METHODS

The study was approved by the medical ethical committee of the Radboud University Nijmegen Medical Centre. Written informed consent was obtained from all participants prior to enrollment.

### Participants

Patients were recruited through the outpatient clinic of the Department of General Internal Medicine of the Radboud University Nijmegen Medical Centre and through an advertisement in the newsletter of the Dutch CFS patient organization ME-Stichting (http://www.me-cvs-stichting.nl). Patients were eligible for participation if they fulfilled the following inclusion criteria: they had to be between 18 and 65 y of age, and they had to fulfill the CDC consensus criteria for CFS [1]. A Structured Clinical Interview for DSM-IV (SCID-1) was performed to exclude patients with current psychiatric co-morbidity and to ensure a homogeneous group of patients.

Pregnant or lactating women were excluded, as were patients with lactose intolerance and patients taking psychotropic drugs or experimental medications.

An additional criterion to be met was an IGFBP3/IGF1 ratio greater than 2.5. We asked patients who could potentially participate in the trial, if possible, to bring a healthy neighborhood control individual of similar age, gender, body weight, and body height, to compare IGF status. Except for contraceptives, the control individuals were not allowed to take medication.

### Interventions

Acclydine and an identical placebo were delivered by the manufacturer Optipharma. Each Acclydine capsule contained 250 mg of the alkaloid. Patients took a single daily dose on an empty stomach, with the following decreasing Acclydine dosage schedule over a total of 14 wk: weeks 1–2, 1,000 mg per day; weeks 3–6, 750 mg per day; weeks 7–8, 500 mg per day; weeks 9–10, 500 mg every 2 d; weeks 11–12, 250 mg per day; and weeks 13–14, 250 mg every 2 d.

Acclydine treatment was combined with amino acid supplements to provide sufficient essential and nonessential amino acid intake during treatment. Patients in the placebo group received placebo Acclydine and placebo amino acid supplements.

There was no difference in taste, appearance, or packaging between the active supplements and the placebo capsules.

### Design and Procedures

The study was a prospective, randomized, double-blind, placebo-controlled trial. The effect of Acclydine was assessed by pre- and post-trial testing. Post-trial testing was performed at the end of the 14-wk treatment period. During the post-trial assessments, the patients were still taking the trial supplements. All participants, investigators, and laboratory technicians were blinded to the treatment condition. IGF1 and IGFBP3 measurements were also done in a blinded fashion.

### Randomization

Before the start of the clinical trial, the pharmacy of the hospital prepared 57 treatment packages. Randomization and allocation to the treatment or placebo group was based on a patient's study number. After acceptance of a patient by the junior researcher (G. K. H. T.) and the clinical psychologist (G. B.), the eligible patient received the lowest study number available (1–57). The pharmacy held the randomization list that correlated the study number with the treatment group. To maintain balance over time, the concealed randomization was done in blocks of ten. Treatments were generated randomly within the blocks using a computer program (Excel, Microsoft, http://www.microsoft.com).

### Primary Outcome Measures

#### Fatigue severity.

The Checklist Individual Strength is a reliable and validated self-report questionnaire. We used the Checklist Individual Strength, subscale fatigue severity (CIS-fatigue) [15,16]. The score on this eight-item scale ranges from eight (no fatigue) to 56 (maximally fatigued). The cut-off point was set at 35 [17].

#### Functional impairment.

The Sickness Impact Profile-8 (SIP-8) measures the influence of symptoms on daily functioning, using the following eight subscales to rate both physical and psychological disability: home management, mobility, alertness behavior, sleep/rest, ambulation, social interactions, work, and recreation and pastimes [18,19]. CFS patients with substantial functional impairments were included. Patients with scores above the cut-off point of 800 were included.

### Secondary Outcome Measures

#### Activity level.

Besides self-reported outcome measures, we measured physical activity with an actometer. An actometer is a small motion-sensing device attached to the ankle; it was worn continuously for 14 d during the assessment periods. After the 2-wk period, the average score over 12 d was computed [20].

#### Daily fatigue level.

During the 2-wk actometer period, patients rated their fatigue level in a complaint diary. They rated their level of fatigue four times a day on a zero (no fatigue) to four (maximally fatigued) scale. The four scores for each day were summed to produce the daily observed fatigue (DOF) score, which ranged from 0 to 16 [21]. The mean of 12 consecutive DOF scores was used.

### Blood Samples

Blood samples of the patients and the matched neighborhood controls were taken at the same time, and the paired blinded samples were handled in an identical fashion. Serum IGF1 was measured by an immune radiometric assay (Diagnostic Systems Laboratories, http://www.dslabs.com). For IGF1, the inter- and intra-assay coefficients of variation were 6.25% and 4.93%, respectively. IGFBP3 was also assessed by an immune radiometric assay (Immulite, DPC, http://dpcweb.com). For IGFBP3, the inter- and intra-assay coefficients of variation were 4.25% and 1.3%, respectively.

### Statistical Methods

For all analyses SPSS 12.01 (SPSS, http://www.spss.com) was used.

Power calculations before the start of the trial showed that 22 persons were needed in each group to detect a difference of at least one standard deviation (SD) on the CIS-fatigue with a power of 90% and a two-tailed significance level of 5%. Anticipating a dropout rate of 10%, at least 49 persons needed to be recruited. Analyses were performed on an intention-to-treat basis.

Missing values were replaced by way of the last observation carried forward. Independent sample *t*-tests were performed on the change scores, defined as the difference between baseline scores and the post-treatment scores after 14 wk.

IGF data are given as mean ± SD. The hormonal measurements were analyzed by independent sample *t*-test.

## RESULTS

All patients were recruited between February 2003 and April 2005.

In total, 22 patients were able to bring a neighborhood control individual (four men and 18 women). The results of the hormonal assessments are shown in Table 1. Hormonal values including the CFS patients of the outpatient clinic and patients who did not meet the hormonal inclusion criteria for participation in the clinical trial are reported.

**Table 1:**
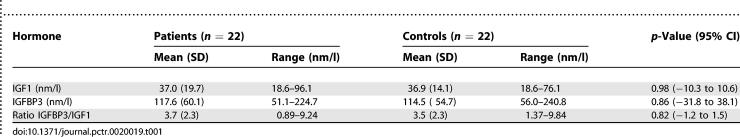
Hormonal Blood Levels

No significant differences were found in the IGF status of the CFS patients versus the controls.

**Figure 1:**
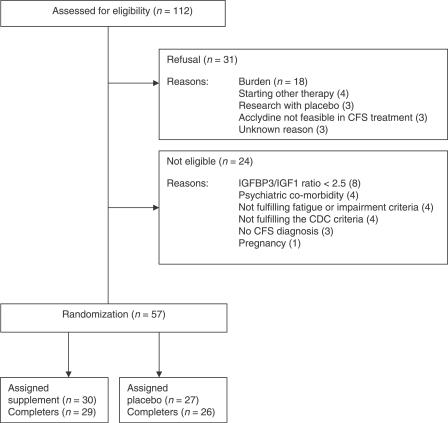
CONSORT Flowchart

Figure 1 illustrates participant flow through the trial. In total, 112 patients were given information about the study protocol; 31 persons refused to participate, and the main reason given for refusal was the intensity of the study. Data obtained from 26 of the 31 persons who chose not to participate showed no significant difference in age, number of CDC symptoms, fatigue severity, or functional impairment between the CFS patients participating in the clinical trial and those who chose not to participate (data not shown). Twenty-four patients did not meet the inclusion criteria. A total of 57 patients were randomized in the clinical trial: 15 patients were recruited from the outpatient clinic, and 42 patients came in response to the advertisement in the patient organization newsletter. One patient in each arm dropped out after randomization, and as a consequence, in each arm, baseline data for one patient was carried forward, and a sensitivity analysis was not required. The Acclydine and placebo treatments were well tolerated. No important side effects were reported in either group. The Acclydine and placebo groups did not differ with respect to age, gender, fatigue severity, impairment, or number of CDC symptoms (Table 2).

**Table 2:**
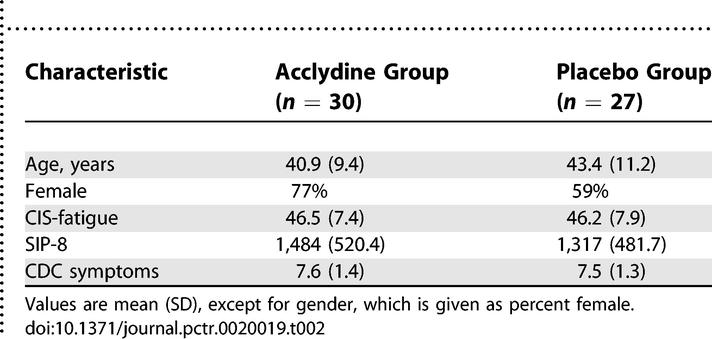
Patient Characteristics

### Primary Outcomes

No significant differences in change scores were found between the treatment and placebo groups on the primary outcome measures (Table 3). The CFS patients treated with Acclydine did not show a significant decrease in fatigue severity (CIS-fatigue +1.1 [95% CI −4.4 to +6.5, *p =* 0.70]) or functional impairment (SIP-8 +59.1 [95% CI −201.7 to +319.8, *p =* 0.65]) compared to the placebo group.

**Table 3:**
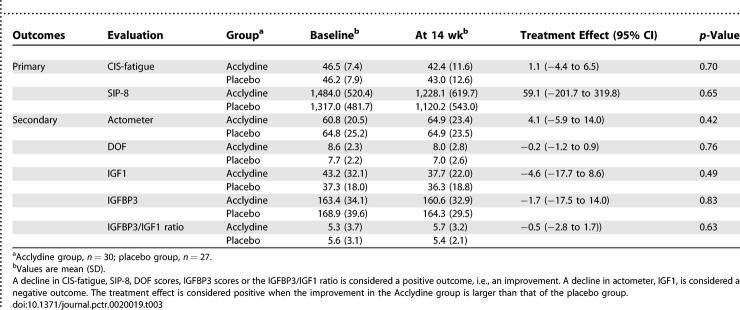
Treatment Effect

### Secondary Outcomes

Actometer activity scores did not show significant differences between the two groups. Analysis of fatigue severity rated with the DOF did not show significant differences either.

**Figure 2:**
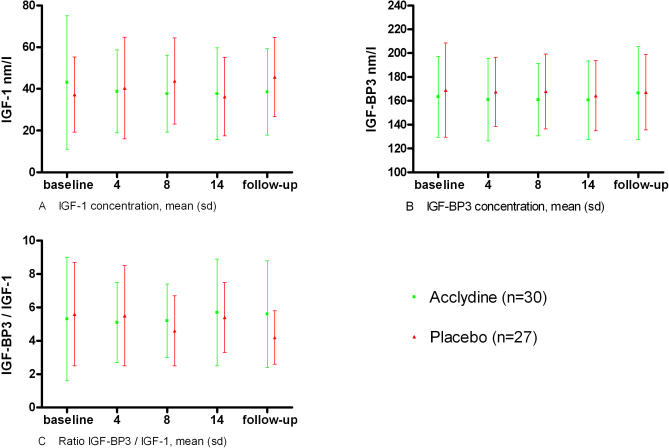
Hormone Data of the Two Treatment Groups (A) IGF1 concentration. (B) IGFBP3 concentration. (C) IGFBP3/IGF1 ratio.

No significant differences were found between the treatment and placebo groups in IGF1 blood level, IBFBP3 blood level, or IGFBP3/IGF1 ratio at baseline; after 4, 8, or 14 wk of treatment; or during follow-up (Figure 2).

## DISCUSSION

### Interpretation

In this randomized controlled blinded clinical trial, no therapeutic effect of Acclydine in CFS was found. In this study we also addressed the issue of a deficiency of bioactive IGF1. To control for unwanted stress effects and other confounders, the blood samples were taken simultaneously from the patients and their matched neighborhood controls. For each pair of patient and control, the measurements of IGF1 and IGFBP3 were performed in the same run. In addition, we detected no differences in IGF1 or IGFBP3 concentrations in CFS patients without psychiatric co-morbidity versus closely matched healthy neighborhood controls.

Although the sample size is relatively small, the results do not point to a different IGFBP3/IGF1 status in CFS patients compared to controls. These results do not support the hypothesis that changes in IGF1 and IGFBP3 metabolism are involved in the pathophysiology of CFS.

### Generalizability

In this study we enrolled a representative sample of adult referred and non-referred CFS patients who fulfilled the international CDC consensus criteria for CFS [1]. In contrast to previous intervention studies with cognitive behavioral therapy [22] or with a polynutrient supplement [23] conducted by our research group, we excluded patients with psychiatric co-morbidity to exclude hormonal influences caused by current psychiatric co-morbidity. The mean fatigue severity and functional impairment scores in this study represent high fatigue severity levels and high functional impairment in the CFS patients. Compared to the previously mentioned intervention studies, the patients in this clinical trial reported slightly lower fatigue and functional impairment scores. Selection of CFS patients without current psychiatric co-morbidity could explain this difference.

### Overall Evidence

To our knowledge, this is the first published report of a randomized controlled trial evaluating the effect of the food supplement Acclydine in CFS patients. The effect was assessed by pre- and post-trial testing with validated instruments designed to assess different dimensions of CFS as well as treatment effects. Treatment with Acclydine was not more effective than placebo in changing self-reported outcome measures, hormonal blood levels, or physical activity.

The lack of significant differences on any of the dimensions of fatigue or the hormonal assessments strengthens our overall findings.

Previous unpublished studies by others on file at the manufacturer claimed that treatment with Acclydine would be more effective in CFS patients with a higher IGFBP3/IGF1 ratio. Based on this claim, we did a subset analysis for patients with a ratio greater than 4.5:19 CFS patients in the Acclydine group and 20 in the placebo group had a ratio greater than 4.5. No significant differences in change scores were seen between the subgroups in any of the outcome measures.

A second claim was that Acclydine treatment works by increasing biologically active IGF1. However, assessment of IGF1 concentration and IGFBP3/IGF1 values in the Acclydine group at baseline, 4 wk, and 8 wk during treatment, at the end of treatment, and at follow-up (1 mo after the treatment period) did not show a significant change.

The power of our study was sufficient to detect changes in time in both groups. Thus, the negative findings found in this randomized controlled trial cannot be explained by a power problem. Considering the reported treatment effect on the primary outcome measures, we believe it is very unlikely that a larger trial would detect a clinically meaningful effect.

It is of interest to note that we detected a minor placebo effect in this study. This is in accordance with observations from earlier randomized controlled clinical trials in CFS [23–25]. However, the minimal decline in fatigue severity and functional impairment during pre- and post-trial testing in both treatment groups could also be partly explained by the phenomenon regression to the mean or a Hawthorne effect.

We did not monitor patient compliance on a daily basis. During the trial, patients had an appointment by telephone or at the outpatient clinic every 2 wk. During these appointments, we assessed whether the capsules were taken as directed, and the treatment protocol for the next 2 wk was discussed with the patient. Although adherence could not be verified fully, there is no reason to believe that lack of adherence can explain the negative findings.

In conclusion, this randomized controlled trial did not demonstrate an effect of Acclydine on biologically active IGF1, nor did it demonstrate any benefit in CFS-related outcome measures. Thus, the findings of this clinical trial do not support the use of Acclydine in the treatment for CFS.

We feel that the negative results of this trial are important for two reasons: Acclydine is expensive, so patients might be spending a lot of money on an ineffective treatment, and it is also available without prescription on the Internet, making it available to patients potentially without a doctor's oversight.

## Supporting Information

CONSORT Checklist Click here for additional data file. (58 KB DOC)

Trial Protocol Click here for additional data file. (262 KB DOC)

Alternative Language Abstract S1 Translation of Abstract into German by Andres How Ming Neuhaus (12 KB DOC)

Alternative Language Abstract S2 Translation of Abstract into Chinese by Yong Xue (162 KB PDF)

## Abbreviations

CDC: US Centers for Disease Control and Prevention
CI: confidence interval
CFS: chronic fatigue syndrome
CIS-fatigue: Checklist Individual Strength, subscale fatigue severity
DOF: daily observed fatigue
GH: growth hormone
IGF: insulin-like growth factor
IGFBP: insulin-like growth factor binding protein
SD: standard deviation
SIP-8: Sickness Impact Profile-8
