# Supplementary material for: The Effect of Acclydine in Chronic Fatigue Syndrome: A Randomized Controlled Trial
Source: PLoS Clin Trials. 2007 May 18;2(5):e19. doi: 10.1371/journal.pctr.0020019 (PMC1876596; doi:10.1371/journal.pctr.0020019)
Supplement: Trial Protocol [file pctr.0020019.sd002.doc]

# **PROTOCOL**

###

#### HET EFFECT VAN ACCLYDINE

#### GECOMBINEERD MET AMINOZUREN OP DE

**VERMOEIDHEID EN BEPERKINGEN**

**BIJ CVS-PATIËNTEN**

## Introductie

Chronisch vermoeidheidssyndroom (CVS), in de volksmond ook ME genoemd, wordt gekenmerkt door ernstige invaliderende vermoeidheid. Patiënten met CVS hebben minimaal zes maanden bestaande ernstige vermoeidheid, die leidt tot ernstige beperkingen in het dagelijks functioneren en waarvoor geen somatische verklaring gevonden kan worden[[1]](#endnote-2).

Heden zijn er in Nederland ongeveer 30.000 CVS-patiënten. Bijna een op elke vijfhonderd Nederlanders lijdt aan het chronisch vermoeidheidssyndroom. De geschatte incidentie ligt rond de 6000 patiënten per jaar. Van de patiënten is acht procent jonger dan achttien jaar. De verhouding mannen-vrouwen is 1:4[[2]](#endnote-3).

CVS is een maatschappelijk probleem. De medische consumptie van CVS-patiënten is hoog en de geschatte maatschappelijke kosten van CVS liggen tussen de 560 miljoen en 1.34 miljard gulden per jaar[[3]](#endnote-4).

CVS wordt gezien als een multifactoriële aandoening waarbij zowel psychologische als somatische factoren een rol kunnen spelen.

Heden is de enige aantoonbaar werkzame behandeling cognitieve gedragstherapie voor de grote groep CVS-patiënten[[4]](#endnote-5).

Het verrichten van verder onderzoek is van groot belang.

## Achtergrond IGF-1/GH

###### IGF-1

Insuline-like Growth Factor-1, voorheen ook wel somatomedine-C genoemd, is een klein polypeptide met een molecuulgewicht van 7,5 kDa. IGF-1 behoort tot een groep mediatoren, die naast insuline-achtige metabole effecten een belangrijke rol spelen bij de celproliferatie en differentiatie.

IGF-1 wordt in veel organen, waaronder de lever, onder invloed van insuline gesynthetiseerd en uitgescheiden in de circulatie. Ongeveer 1 tot 5 procent van het totaal IGF-1 circuleert ongebonden in het bloed.

In de circulatie is het IGF-1 gebonden aan verschillende eiwitten, waarvan IGF-BP-3 (IGF-binding-protein-number-3) het belangrijkste is. Deze bindende eiwitten vervullen een transportfunctie en verlengen de verblijfsduur van IGF-1 in het plasma.

De plasma IGF-1 concentratie staat, evenals de concentratie van IGF-BP-3, onder invloed van groeihormoon (GH), maar wordt daarnaast ook beïnvloed door een groot aantal andere factoren zoals leeftijd, geslacht, voedingstoestand en de concentratie van diverse andere hormonen, waaronder insuline, thyroxine, geslachtssteroïden, corticosteroïden en prolactine. De plasmaconcentratie is overdag min of meer stabiel, zodat in de praktijk geen rekening hoeft te worden gehouden met het tijdstip van bloedafname. Vanaf de geboorte stijgt het IGF-1 gehalte in de circulatie en rond de maximale groeisnelheid, tijdens de puberteit, wordt de hoogste waarde bereikt. Het leeftijd-afhankelijke-patroon van de IGF-1-secretie komt overeen met dat van de GH-secretie. Na de puberteit nemen de plasmaspiegels van IGF-1 en GH geleidelijk af[[5]](#endnote-6).

## GH

Groeihormoon (GH), ook wel somatotroop hormoon (STH) of somatotropine genoemd, is een polypeptide. Het wordt gevormd in de eosinofiele cellen van de hypofysevoorkwab onder invloed van het GH releasing hormone (GH-RH). De adenohypofyse bevat ca. 1000 maal zoveel groeihormoon als de overig hormonen. Dagelijks wordt ongeveer 5% van het aanwezige hormoon afgescheiden; de afgifte kan echter van moment op moment sterk verschillen.

GH heeft een lange- en korte-termijnwerking. Het effect van GH wordt gemedieerd door somatomedines, ook wel aangeduid als IGF. De belangrijkste groeifactor is IGF-1.

Via IGF-1 wordt de opname van aminozuren in de cellen en de eiwitsynthese gestimuleerd. Als zodaning bevordert het naast andere bouwstofwisselings-effecten, de lengtegroei bij jonge mensen.

Het korte-termijneffect kan worden aangeduid met een aanpassing aan vasten.

Daling van de glucoseconcentratie in het bloedplasma verhoogt de de GH-release. GH mobiliseert vet en glycogeen en vermindert de glucose-opname in de cellen. De glucosespiegel in het bloed stijgt en de vetstofwisseling neemt toe, waardoor vetafbraakprodukten in het bloed verschijnen. De stijgende glucosespiegel remt op zijn beurt weer de GH-release[[6]](#endnote-7).


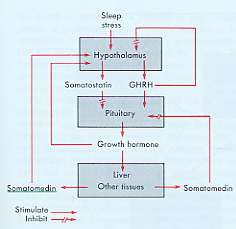


Regulation of GW secretion. The hypothalamic peptide peptide (GHRH) stimulates growth hormone release, whereas the hypothalamic peptide somatostatin inhibits it.

*Negative feedback is by the peripheral mediator of HGC action: somatomedin.*

*Negative feedback occurs both via somatomedin inhibition of GHRH action and by somatomedin stimulation of somatostatin Release.*

*HGH inhibits its own secretion by short-loop feedback. In addition GHRH inhibits its own release via ultra short-loop Feedback. In both of these cases the negative feedback is probably via increasing somatostatin release.*


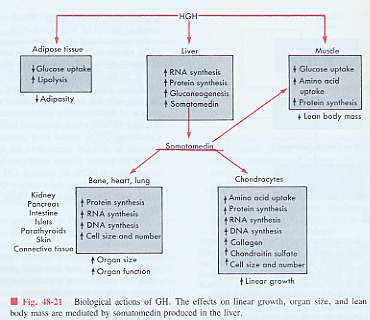
 *Berne RM, Levy MN. Physiologie; third edition:918*

## IGF-1/GH en vermoeidheid

Bij personen met het chronisch vermoeidheidssyndroom heeft volgens sommige onderzoekers 60-70% een verlaagde IGF-1 concentratie[[7]](#endnote-8). Bij deze CVS-patiënten vindt men een verhoogde IGF-BP-3/IGF-1 ratio. Deze ratio maakt deel uit van één van de inclusiecriteria in deze trial.

Meerdere publicaties wijzen in de richting van verlaagde IGF-1 concentraties bij CVS-patiënten[[8]](#endnote-9) [[9]](#endnote-10). Moorkens et al. melden verminderde nachtelijke groeihormoon (GH) – secretie en lage IGF-1 concentraties[[10]](#endnote-11).

IGF-1 speelt een belangrijke rol in het metabolisme van een persoon. Zoals reeds beschreven stimuleert IGF-1 de metabolisme in de skeletspier; het bevordert de opname van aminozuren in de skeletspier en draagt hiermee bij aan een positieve stikstofbalans. Een toename van de IGF-1 leidt tot een verhoogde eiwitopbouw van de cel, stimulering van de vetstofwisseling en stimulering van de energiestofwisseling[[11]](#endnote-12).

Uit onderzoek zijn aanwijzingen gevonden dat de IGF-1 concentratie gerelateerd is aan de vermoeidheid en het energieniveau van een persoon[[12]](#endnote-13).Verder zijn positieve correlaties aangetoond dat hogere biologisch actieve IGF-1 concentratie gerelateerd is aan een beter fysiek prestatievermogen[[13]](#endnote-14).

**Acclydine-aminozuren kuur / IGF-1**

Acclydine is een voedingssupplement op plantaardige basis ontwikkeld. Het is vrij verkrijgbaar bij de apotheek. Het werkzame bestanddeel GCR is een alkaloïd afkomstig uit solanum dulcamara en verhoogt de plasma IGF-1 concentratie, zoals uit dierexperimenteel en humaanonderzoek blijkt.

De energievoorziening en energieniveau van een persoon is afhankelijk van de energieproductie in de cellen. Voor een goede energieproductie zijn een goede aanvoer van bouwstenen nodig zoals, glucose, aminozuren en vetten naar de lichaamscellen.

Acclydine zou de opname daarvan bevorderen en op deze manier de energieproductie in de cellen stimulere. Acclidine zou op cellulair niveau de IGF-1 receptoren, de tyrosine kinase receptoren en de integrinreceptoren stimuleren en de hypothalamus de secretie van Groeihormoon Releasing Hormoon verhogen. Dit leidt tot een verhoogde afgifte van groeihormoon door de hypofyse.


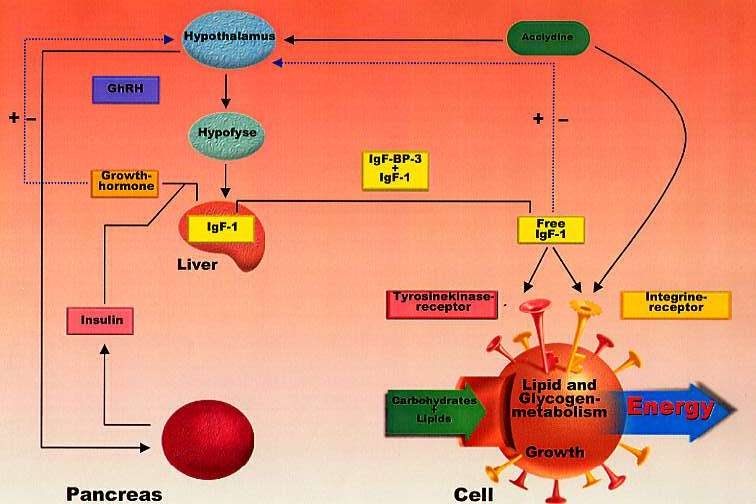


## Aminozuren suppletie

Samen met Acclydine maken ‘All-Amino S’en ‘Glutaminol’ deel uit van de behandeling.

Aminozuren suppletie zou nodig zijn voor een evenwichtige en vrij constante aanvoer van aminozuren plaats naar de lichaamscellen.

*All-Amino S*. bevat voldoende essentiële en niet-essentiële aminozuren voor de dagelijkse behoefte en is bovendien versterkt met een aantal vrije aminozuren (zie bijlage voor samenstelling).

*Glutaminol, glutamine* is een veelzijdig aminozuur waarvan de consumptie door het maag -darmkanaal, immuniteitscellen en de nieren toeneemt in perioden van stress. Bij metabole stress neemt de glutamineproductie door spierweefsel (het belangrijkste glutamineproducerende orgaan) toe[[14]](#endnote-15). In een later stadium leidt dit tot een daling van de plasma-glutamine –concentratie. De behoefte van deze weefsels kan in die omstandigheden de productiecapaciteit van het lichaam overstijgen, waardoor een tekort optreedt. Aanvulling in zulke gevallen verbetert de conditie van de maag en de darmen en verhoogt de immuniteit[[15]](#endnote-16).

Glutaminol kan dienen als alternatieve energiebron voor de hersenen naast glucose.

Een verbeterde ‘energieflow’ zou de geestelijke vermoeidheid verminderen en de alertheid en concentratie verbeteren.

In een onderzoek in Brussel zouden indrukwekkende resultaten bereikt zijn met acclydine en de aminozuur suppletie, met name bij die patiënten die een ratio IGF-BP-3/IGF->4.5 hadden.

Gezien de hiervoor beschreven resultaten en fysiologie wil de onderzoeksgroep CVS in het UMC St. Radboud de effectiviteit van het voedingssupplement Acclydine gecombineerd met aminozuren onderzoeken bij CVS-patiënten. Mogelijk heeft een therapie dat een verhoging van de plasma IGF-1 concentratie bewerkstelligd een gunstig effect op de CVS gerelateerde klachten.

## Doelstelling

1. Onderzoek naar het effect van Acclydine gecombineerd met aminozuren op de vermoeidheid en beperkingen bij CVS-patiënten.
2. Onderzoek naar de bestendigheid van de mogelijke effecten bij CVS-patiënten na het staken van de medicatie.
3. Onderzoek naar de effecten van Acclydine op de IGF-1 en IGF-BP-3 concentraties bij CVS-patiënten.

## Experimentele opzet

Type studie: Dubbel blind placebo gecontroleerde studie

Aantal patiënten: 2 x 30 patiënten.

Studieduur: 18 weken

Supplementatie duur: 14 weken.

Doseringen: Acclydine kuur – zie bijlage.

Placebo

Randomisatie: Het randomisatie-schema zal worden opgesteld door de apotheek.

Uitkomstmaten

Primair: Ernst van de ervaren vermoeidheid: CIS-fatigue.

Beperkingen: SIP-8

Stijging van de IGF-1 concentratie.

Daling van de IGF-3 concentratie.

Effect modifier Activiteiten niveau: Actometer.

Poli-contacten : 9 maal. Zie studie design.

Bloedbepaling: 4 meetmomenten.

###### Inclusie-rate 4 patiënten per week.

###### Meetinstrumenten

###### Gevalideerde vragenlijsten

Ernst van de vermoeidheid

* Checklist Individual Strength (CIS). De CIS is een betrouwbare en gevalideerde vragenlijst**[[16]](#endnote-17)**. De CIS meet het vermoeidheidsbeeld over de twee voorafgaande weken, waarin fluctuaties in de tijd meegenomen worden. Vier facetten van vermoeidheid worden gemeten:

- ondervonden moeheid, acht items
- concentratie, vijf items
- motivatie, vier items
- subjectief gerapporteerde acitviteiten-niveau, drie items

In totaal bestaat de CIS uit twintig korte uitspraken of items. Elk item wordt op een 7-punts Likert schaal gescoord. Hoge totaalscores betekenen een hoog vermoeidheidsniveau , een hoge mate van concentratieproblemen, lage motivatie en een laag niveau van lichamelijke activiteit.

De subschaal moeheid (CIS-moe) bestaat uit acht vragen. De score varieert van 8 tot 56. Een score van 40 of hoger is gehanteerd als indicatie voor ernstige vermoeidheid en tevens als inclusiecriterium.

***** Zelfobservatielijst: De vermoeidheid (0-4) zal op 4 tijdstippen per dag worden ingevuld gedurende 12 dagen. De variabele dagelijks moeheid (dag-moe) is het gemiddelde van de somscores per dag (range 0-16) (Vercoulen et al., 1996).

Functionele beperkingen

* Sickness Impact Profile (SIP). De aard en mate van beperkingen bij CVS- patiënten kunnen worden vastgesteld met behulp van de SIP. Op basis van eerder CVS onderzoek zijn 8 van de 12 subschalen gebruikt (SIP-8). De acht subschalen zijn: slapen/rusten, huishouden, mobiliteit, sociale interacties, lopen, alertheid, werk en recreatie/vrije tijd. De SIP-8 bestaat uit 86 uitspraken, elk voorzien van een wegingsfactor. De mate van beperkingen is bepaald door de som van de subschaalscores. Een score van 800 of hoger is gehanteerd als indicatie voor ernstige beperkingen en als inclusiecriterium.

Lichamelijke activiteit

***** Actometer. Dit kleine apparaat wordt gedurende 12 dagen dag en nacht om de enkel gedragen om het niveau van lichamelijke activiteit te meten. De actometer telt het aantal bewegingen per periode van vijf minuten. Deze informatie wordt opgeslagen in een intern geheugen en kan worden uitgelezen met behulp van speciaal ontwikkelde software. Per dag wordt een gemiddeld dagscore berekend. Het gemiddelde van de 12 dagscores weerspiegelt het gemiddelde activiteitenniveau (actometer-mean). Hoe hoger de score, des te actiever de patiënt is geweest.

Voor elke patiënt is tevens het activiteitenpatroon berekend, door de 12 dagscores te vergelijken met een referentiewaarde, gebaseerd op het gemiddelde van verschillende groepen CVS-patiënten. Drie verschillende activiteitenpatronen kunnen bij CVS-patiënten worden onderscheiden: voortdurend passief, wisselend actief en voortdurend actief[[17]](#endnote-18).

## Studie populatie

Nieuwe patiënten die op de poli algemeen interne geneeskunde gediagnosticeerd worden met CVS en schriftelijk aangegeven hebben interesse te hebben in wetenschappelijk onderzoek worden benaderd.

#

Patiënten zullen worden geïnformeerd over de studie.

CVS-patiënten met een ratio IGF-BP-3/IGF-1>4.5 en een bepaalde score op de twee test-scores ( zie inclusiecriteria), kunnen worden geïncludeerd.

##### Inclusiecriteria

- CVS-patiënten; CDC-criteria
- Mannelijk en vrouwelijke patiënten: leeftijd tussen de 18 en 65 jaar.
- Een verhoogde IGF-BP-3/IGF-1 ratio>2.5.
- CIS-score >35 subschaal fatigue severity.
- SIP-8-totaalscore >800
- (schriftelijk) Informed consent.

Exclusiecriteria

- Zwangerschap, of de intentie tot een zwangerschap.
- Vrouwen die borstvoeding geven.
- Deelname aan andere therapieën.
- Eerdere participatie aan recente clinical trials.
- Patiënten met actuele psychiatrische co-morbiditeit
- Patiënten met co-morbiditeit waarvoor zij medicamenteus worden behandeld.

## Bloedbepaling

De hormoonbepalingen worden door de firma DSL worden uitgevoerd.

De ingestuurde sera kunnen niet naar de patiënt worden herleid. Op de verschillende bloedbuisjes zal alleen een nummer worden vermeld.

Door middel van Elisa-techniek worden IGF-1 en IGF-BP-3 bepaald.

# De IGF-1 bepalingen hebben een intra –en intervariabiliteit van beide 4%. De IGF-BP-3 bepalingen hebben een intra –en intervariabiliteit van 6.8%.

**Risico’s**

Gerapporteerde bijwerkingen van Accydine zijn hoofdpijn en misselijkheid.

Bij inname van Acclydine op de nuchtere maag, kan een persoon zich soms misselijk voelen. Om deze reden wordt u geadviseerd de voedingssupplementen één uur na de maaltijd te nemen. Een ander gerapporteerde bijwerking is hoofdpijn. Als hoofdpijn optreedt als bijwerking, komt deze meestal voor bij aanvang van de kuur. De mogelijke bijwerkingen zijn in het algemeen licht of matig van aard. Overgevoeligheidsreacties zijn niet gemeld.

In de placebo-tabletten bevindt zich koemelk eiwit. Wanneer een patiënt hiervoor allergisch is kan hij bijvoobeeld reageren met een huiduitslag.

## Onafhankelijke arts

Dr. J.W.M Lenders – internist

Afdeling: Algemeen Interne Geneeskunde

Tel: 024-3676851

Tel secretariaat: 024-3614763

## Studie design

Het onderzoek bestaat uit 9 poli-contacten van een uur.

Tijdsduur: Totaal 18 weken (zie schema).

**Polibezoek-1.**

Studie opzet:

Bij het eerste contact zal de opzet van de studie worden uitgelegd.

Mede zal worden uitgelegd dat deelname aan het onderzoek afhangt van de bloeduitslag en het intakegesprek.

Bloedbepaling:

Bloed zal worden afgenomen ten behoeve van het inclusie criterium: een verhoogde ratio IGF-BP3/IGF-1>4.5. Tevens wordt het verdere bloedbeeld bepaald ter controle van de andere bloedbeeld-criteria.

Testorganisor:

De patiënten wordt gevraagd de verschillende vragenlijsten in te vullen.

## Polibezoek-2.

Intakegesprek:

Tijdens het tweede contact zal de intakegesprek plaatsvinden.

Wanneer het bloedbeeld voldoet aan de voorafgestelde criteria zal een psycholoog van de afdeling medische psychologie de verdere inclusie –en exclusiecriteria toetsen.

Start supplementatieperiode

Na inclusie en randomisatie zal gestart worden met de supplementatieperiode.

De Acclydine-supplementatie of de placebo zal worden uitgereikt voor een periode van twee weken.

**Polibezoek-3.**

- - Begeleiding / nagaan van compliance en mogelijke bijwerkingen.
  - Testorganisor.
  - Uitreiken Acclydine of placebo.

**Polibezoek-4**

- - Begeleiding / nagaan van compliance en mogelijke bijwerkingen.
  - Testorganisor.
  - Uitreiken Accydine of placebo.
  - Bloedonderzoek.

**Polibezoek-5.**

- - Begeleiding / nagaan van compliance en mogelijke bijwerkingen.
  - Testorganisor.
  - Uitreiken Acclydine of placebo.

**Polibezoek-6.**

- - Begeleiding / nagaan van compliance en mogelijke bijwerkingen.
  - Testorganisor.
  - Uitreiken Accydine of placebo.
  - Bloedonderzoek.

**Polibezoek-7.**

- - Begeleiding / nagaan van compliance en mogelijke bijwerkingen.
  - Testorganisor.
  - Uitreiken Acclydine of placebo.
  - Start actometerperiode.

**Polibezoek-8: Eind supplementatieperiode.**

- - Begeleiding / nagaan van compliance en mogelijke bijwerkingen.
  - Testorganisor.
  - Bloedonderzoek.
  - Inname actometer.

**Polibezoek-9: Nameting.**

- - Nagaan van veranderingen na staken van de medicatie.
  - Evaluatie onderzoek met patiënt.
  - Testorganisor.
  - Bloedonderzoek.

**Schema Acclydinekuur**

**Week 1+2:** ’s morgens 2 Acclydine ’s avonds 2 Acclydine

2 Glutaminol 2 Glutaminol

5 All-Amino-S 6 All-Amino-S

**Week 3+4:** ’s morgens 2 Acclydine ’s avonds 1 Acclydine

2 Glutaminol 2 Glutaminol

5 All-Amino-S 3 All-Amino-S

**Week 5+6:** ’s morgens 2 Acclydine ’s avonds 1 Acclydine

2 Glutaminol 2 Glutaminol

5 All-Amino-S 3 All-Amino-S

**Week 7+8:** ’s morgens 1 Acclydine ’s avonds 1 Acclydine

2 Glutaminol 2 Glutaminol

2 All-Amino-S 1 All-Amino-S

**Week 9+10:** ’s morgens 1 Acclydine ‘s avonds

2 Glutaminol 1 Glutaminol

2 All-Amino-S 1 All-Amino-S

**Week 11+12:** oneven 1 Acclydine

1Glutaminol

2 All-Amino-S

even 1 Glutaminol

1 All-Amino-S

**Week 13+14** ’s morgens en ’s avonds 1 Glutaminol 1 All-Amino-S

1. Fukuda K, Straus SE, Hickie I, Sharpe MC, Dobbins JG, Komaroff A & the Chronic Fatigue Syndrome Study Group. The chronic fatigue syndrome: a comprehensive approach to its definition and study.

   Annals of Internal Medicine 1994;121:953-9. [↑](#endnote-ref-2)
2. Bazelmans E, Vercoulen J, Galama J, van Weel C, van der Meer J, Bleijenberg G. Prevalence of chronic fatigue syndrome and primary fibromyalgia syndrome in the Netherlands. Ned Tijdschr Gen. 1997;141,31: 1520-1523. [↑](#endnote-ref-3)
3. Burgering C. Rapport: Maatschappelijke kosten van ME/CVS. Economische en Sociaal Instituut van de Vrije Universiteit (ESI/VU). [↑](#endnote-ref-4)
4. Wessely S. Chronic fatigue syndrome - trials and tribulations. JAMA, 2001; 286, 11:1378-1379. [↑](#endnote-ref-5)
5. Diagnostisch Kompas, College voor zorgverzekeringen.1999/2000; 560-561. [↑](#endnote-ref-6)
6. Bernards et al., Fysiologie van de mens. [↑](#endnote-ref-7)
7. Keizer HA, Costongs GM, Helden J. Een mogelijk nieuwe therapie voor patiënten met het chronisch vermoeidheidssyndroom. (submitted for publication). [↑](#endnote-ref-8)
8. Allain T, Bearn J, Coskeran P. Changes in growth hormone, insulin, insulin-like growth factors (IGFs), and IGF-binding protein-1 in chronic fatigue syndrome. Biol Psychiatriy 1997;41:567-573. [↑](#endnote-ref-9)
9. Berwaerts, J., Moorkens, G., Abs, R.. Secretion of growth hormone in patients with chonic fatigue syndrome. Growth Hormone IGF Res.,1998;8 Suppl B:127-129. [↑](#endnote-ref-10)
10. Moorkens, G., et al.. Characterization of pituitary function with emphasis on GH secretion in the chronic fatigue syndrome. Clin. Endocinology, 2000;53(1):96-106. [↑](#endnote-ref-11)
11. Salomon F, Cuneo R, Sonksen P. Growth hormone and protein metabolism. Horm Res, 1991;36

    (suppl 1): 41-41 [↑](#endnote-ref-12)
12. Breda van E, Rietjens G, Keizer H. Effects of a new food supplement on serum Insulin-Like Growth Factor-1 (IGF-1) and Insulin-Like Growth Factor Binding Protein-3 (IGF-BP-3) concentrations during rest and exercise. Submitted for publication. [↑](#endnote-ref-13)
13. Chadan, S.G., et al.. Influenxe of physical activity on plasma insulin-like growth factor-1 and insulin-like growth factor binding proteins in healthy older women. Mech. Ageing Dev. 1999;109(1):75-83. [↑](#endnote-ref-14)
14. Windmiller H, Spaeth A. Uptake and metabolism of plasma glutamine by the small intestine. J Biol Chem.,1974;249:5070-5079. [↑](#endnote-ref-15)
15. Stehle P et al. Effect of parenteral glutamine peptide supplements on muscle glutamine loss and nitrogen balance after major surgery. Lancet 1989;1:231-233. [↑](#endnote-ref-16)
16. Vercoulen J, Alberts M, Bleijenberg G. De checklist Individual Strengt (CIS). Gedragstherapie. 1999;32(2): 131-136 [↑](#endnote-ref-17)
17. Werf van der S, Prins J, Vercoulen J, Meer van der J, Bleijenberg G. Identifying physical activity patterns in chronic fatigue syndrome in chronic fatigue syndrome using actigraphic assessment. J. Psychosomatic Research 2000;49,373-379. [↑](#endnote-ref-18)
