# Supplementary material for: The Effect of Acclydine in Chronic Fatigue Syndrome: A Randomized Controlled Trial
Source: PLoS Clin Trials. 2007 May 18;2(5):e19. doi: 10.1371/journal.pctr.0020019 (PMC1876596; doi:10.1371/journal.pctr.0020019)
Supplement: Alternative Language Abstract S1 — (12 KB DOC) [file pctr.0020019.sd003.pdf]

Die Wirkung von Acclydine beim chronischen Fatigue-Syndrom: Eine randomisierte, kontrollierte Studie

G.K.H. The, G. Bleijenberg, J.W.M. van der Meer

Klinik für Innere Medizin, Nijmegen Expert Centre Chronic Fatigue, Medizinisches Zentrum der Radboud Universität, Nijmegen, Niederlande. Postfach 9101, 6500 HB Nijmegen, Niederlande, Tel.: +31 (0)24-3618819, Fax: +31 (0)24-3541734, e-mail: g.the@aig.umcn.nl

Zusammenfassung

Ziele: Es ist unklar, ob Insulin-like Growth Factor (IGF) funktionell an der Pathophysiologie des Chronischen Fatigue-Syndroms (CFS) beteiligt ist. Unveröffentlichte Daten sowie Berichte in Publikationsorganen von Betroffenengruppen suggerieren, dass Acclydine, ein Nahrungsergänzungsmittel, in der Behandlung von Patienten mit CFS durch Erhöhung des physiologischen Plasmaspiegels von IGF-1 wirksam sein könnte. Unseres Wissens wurden bislang keine "peer reviewed", randomisierte, kontrollierte Studien, welche die Wirksamkeit von Acclydine untersuchen, veröffentlicht. In dieser Studie untersuchten wir den Status von IGF-1 und IGF-BP3 bei CFS-Patienten im Vergleich mit nach Alter und Geschlecht vergleichbaren Kontrollen aus der Gemeinde und erfassten die Wirkung von Acclydine auf die Schwere des Fatigue-Syndroms, auf funktionelle Einschränkungen sowie auf den biologisch aktiven Plasmaspiegel von IGF-1 (Ratio IGF-BP3: IGF-1).

Untersuchungsaufbau: Diese Studie war eine randomisierte, Placebo-kontrollierte, doppelblinde klinische Untersuchung.

Studienzentrum: Die Untersuchung wurde am Medizinischen Zentrum der Radboud Universität Nijmegen, Niederlande, durchgeführt.

Teilnehmer: Im Rahmen dieser Studie wurden 57 volljährige Patienten untersucht, welche die CDC-Kriterien für CFS erfüllten. Der IGF-Status von 22 CFS-Patienten wurde mit dem von 22 gesunden und hinsichtlich Alter und Geschlecht angeglichenen Kontrollen aus der Gemeinde stammten waren verglichen.

Interventionen: Die Teilnehmer erhielten Acclydine oder Placebo über jeweils 14 Wochen.

Zielvariablen: Zielvariablen waren die Schwere des Fatigue-Syndroms („Checklist Individual Strength", Subskala Schwere des Fatigue-Syndroms [CIS-fatigue]), die funktionale Beeinträchtigung („Sickness Impact Profile-8" [SIP-8]) sowie der Plasmaspiegel von IGF-1. Die Wirksamkeit von Acclydine wurde mit einer Intention-to-treat-Analyse erfasst. Die Unterschiede zwischen Acclydine- und Placebo-Gruppe hinsichtlich der Veränderungen der Zielvariablen über den 14-wöchigen Zeitraum wurden mit t-Tests für unabhängige Stichproben, Konfidenzintervall (KI) 95%, berechnet.

Resultate: Es gab keinen Unterschied hinsichtlich des IGF-Plasmaspiegels zwischen den 22 CFS-Patienten im Vergleich mit gesunden alters- und geschlechtsgematchten Kontrollen. Die Behandlung mit Acclydine ergab im Vergleich mit der Placebo-Gruppen keine signifikanten Unterschiede in den Zielvariablen: CIS-fatigue +1.1 (95% KI –4.4 bis 6.5, p=0.70), SIP-8 +59.1 (95% KI –201.7 bis 319.8, p=0.65), Ratio IGF-BP3: IGF-1 +0.5 (95% KI –1.7 bis 2.8, p= 0.63).

Schlußfolgerungen: Wir fanden keine Unterschiede des IGF-1 Status zwischen CFS-Patienten und Kontrollen. Zusätzlich zeigen die Ergebnisse dieser Studie keine Überlegenheit von Acclydine über Placebo bei der Behandlung von CFS.

Versuchsregistrierungnummer: ISRCTN77271661

http://www.controlled-trials.com/ISRCTN77271661
