# Supplementary material for: The Effect of Acclydine in Chronic Fatigue Syndrome: A Randomized Controlled Trial
Source: PLoS Clin Trials. 2007 May 18;2(5):e19. doi: 10.1371/journal.pctr.0020019 (PMC1876596; doi:10.1371/journal.pctr.0020019)
Supplement: Alternative Language Abstract S2 — (162 KB PDF) [file pctr.0020019.sd004.pdf]

用隨機分配對照組比較的臨床實驗來研究 ACCLYDINE 對慢性疲勞綜合徵的治療作用.

G.K.H.The, G. Bleijenberg, J.W.M. van der Meer 荷蘭 Nijmegen 大學醫院, 內科, 及 Nijmegen 慢性疲勞研究中心.

Radboud University Nijmegen Medical Centre, Nijmegen The Netherlands. PO Box 9101, 6500 HB Nijmegen, the Netherlands, tel. : +31 (0)24-3618819, fax: +31 (0)24-3541734, e-mail: g.the@aig.umcn.nl, corresponding author

摘要:

研究目的: 類胰島素生長因子(IGF)在慢性疲勞綜合徵發病機理中的作用目前仍不清楚. ACCLYDINE 是一種食物添加劑. 在一些病人協會的新聞短訊上; 未正式發表的結果表明 ACCLYDINE 或許通過增加 IGF-1 的生物活性而達到對慢性疲勞綜合徵病人的治療作用. 關於 ACCLYDINE 的治療效果, 據我行所知目前尚無隨機分配對照組比較的臨床研究結果在正式雜誌上發表.

本實驗目的:

1) 檢測慢性疲勞綜合徵病人的 IGF-1, IGF-BP3 的水平, 並與所居住社區中相同年齡及性別的 人群做比較(對照組);
2) 評估 ACCLYDINE 對以下指標的影響: 疲勞程度, 功能受累程度, 及 IGF-1 的生物活性 (IGF-BP3 與 IGF-1 的比例).

實驗設計: 隨機分配, 安慰劑對照, 雙盲法.

實驗地點: 此實驗在荷蘭 Nijmegen Radboud 大學醫療中心完成.

受試人群: 符合美國疾病控制中心(CDC)制定的慢性疲勞綜合徵診斷標準的 57 名成年病人參與了本實驗. 將其中 22 名病人和另外 22 名對照組健康人的 IGF 進行了比較.

治療方法: 受試人群服用 ACCLYDINE 或安慰劑, 療程 14 周.

分析方法: 疲勞程度用 CIS 一疲勞程度的標準衡量每個受試者. 功能障礙程度用疾病受累程度測試法(SIP-8). IGF-1 的血清水平用來衡量其生物活性.

全部開始服用了 ACCLYDINE 的受試者都包括在 ACCLYDINE 的效果分析. 獨立樣品 t-檢驗 的統計方法被用來比較 ACCLYDINE 與安慰劑組在用藥前與用藥後 14 周各指標的變化, 其可信區間為 95%.

結果: 22 名病人的 IGF 與對照組相比較, 二者無區別. 服用 ACCLYDINE 的治療組與安慰劑組在以下諸指標中無顯著區別: 治療組: CIS 疲勞程度標準: +1.1 (95% ci – 4.4 ~ 6.5, p=0.70), SIP 功能障礙受累程度: +59.1 (95%ci – 201.7 ~ 319.8, P=0.65), IGF-BP3 與 IGF-1 的比例: +0.5 (95%ci – 1.7 ~ 2.8, p= 0.63)

結論: 慢性疲勞綜合徵病人的 IGF-1 與健康對照組相比較, 我行未發現二者有任何區別. 除此之外, 與安慰劑服用組相比, 服用 ACCLYDINE 的病人未顯示任何療效.

臨床實驗注冊號碼: ISRCTN77271661

網址: http://www.controlled-trials.com/ISRCTN77271661
